# Study on the Phenotypic Diversity of 33 Ornamental *Xanthoceras sorbifolium* Cultivars

**DOI:** 10.3390/plants12132448

**Published:** 2023-06-26

**Authors:** Chengyu Zhou, Huaiyan Wu, Qianqian Sheng, Fuliang Cao, Zunling Zhu

**Affiliations:** 1 College of Landscape Architecture, Nanjing Forestry University, Nanjing 210037, China; 2 Co-Innovation Center for Sustainable Forestry in Southern China, Nanjing Forestry University, Nanjing 210037, China; 3 Jin Pu Research Institute, Nanjing Forestry University, Nanjing 210037, China; 4 Research Center for Digital Innovation Design, Nanjing Forestry University, Nanjing 210037, China; 5 College of Art and Design, Nanjing Forestry University, Nanjing 210037, China

**Keywords:** ornamental *Xanthoceras sorbifolium*, germplasm resources, phenotypic traits, phenotypic diversity

## Abstract

*Xanthoceras sorbifolium*, belonging to the family *Sapindaceae*, has a beautiful tree shape, elegant leaves, large and many brightly colored flowers, and a long flowering duration. This plant is widely applied in gardens. In this study, 33 cultivars of *Xanthoceras sorbifolium* were selected from the perspective of ornamental properties, and their phenotypic traits, such as leaves, flowers, and branches, were measured and analyzed, and their phenotypic diversity was comprehensively evaluated using principal component analysis, in order to investigate the phenotypic diversity characteristics of *Xanthoceras sorbifolium*. The results showed that the genetic diversity index of the qualitative traits varied from 0.14 to 1.50, and that of quantitative traits varied from 1.76 to 2.05. The quantitative traits were more diverse than the qualitative traits. The coefficient of variation of the qualitative traits ranged from 16.90% to 57.96%, and that of quantitative traits ranged from 12.92% to 32.87%. The phenotypic traits of the tested cultivars had relatively rich variation. Furthermore, the level of the phenotypic diversity index of *Xanthoceras sorbifolium* was not consistent with the level of coefficient of variation, indicating large variation and uneven distribution of variation. Through principal component analysis, 17 quantitative characters were extracted into five principal components, with a cumulative contribution rate of 79.82%, representing the primary information on the quantitative characters of ornamental *Xanthoceras sorbifolium* cultivars. The F value of the 33 samples ranged from –2.79 to 1.93, and the comprehensive scores of seven cultivars were greater than 1, indicating that these cultivars had rich phenotypic diversity. Therefore, the screening, development, and utilization of fine germplasm resources of *Xanthoceras sorbifolium* should focus on these cultivars. The 33 cultivars were subsequently clustered into five categories through systematic clustering. The cluster analysis provided references for breeding ornamental *Xanthoceras sorbifolium* cultivars with different utilization values, such as large white flowers, small red flowers, large red flowers, large orange flowers, and double-petaled flowers.

## 1. Introduction

*Xanthoceras sorbifolium*, also known as bluffy papaya, canopy, aragonite, and corolla, belongs to the family *Sapindaceae* [1]. Its seeds are edible, highly nutritious, and rich in oil, making it an ideal biodiesel feedstock [2,3]. It is an excellent flower tree and garden greening tree species with a beautiful tree shape, elegant leaves, large and many flowers, bright flower color, and long flower duration [4]. *X. sorbifolium* possesses high stress resistance, cold resistance, drought resistance, barren resistance, and adaptability. It possesses edible, medicinal, ecological, energy, ornamental, and other properties [5,6,7,8], as well as significant development potential.

Current *X. sorbifolium* research is mainly focused on successful cultivation and variety selection [9,10,11], pharmacological and therapeutic benefits of extracts [12], and extraction and utilization of seed kernel oil [13,14,15], all of which have shown relatively fruitful results. However, as an important ornamental tree species, its phenotypic diversity remains slightly under-researched. Plant phenotypic diversity, arising from the interactions between genes and environment, is an important indicator for determining species diversity [16,17] and has been applied to several plants, such as *Hibiscus syriacus* [18], *Nelumbo nucifera* [19], and *Reseda odorata* [20]. *X. sorbifolium* has been in the wild or semi-wild state for a long time, with rich variation types and a wide variation base [21]. Li et al. [22] combined phenotypic traits of *X. sorbifolium* leaves and branches and physiological indicators, such as chlorophyll and carotenoid contents, and divided the 52 *X. sorbifolium* provenances into four groups. Liu et al. [23] found that the 10 phenotypic traits of *X. sorbifolium* seeds were significantly correlated, among which the variation range of the 100-seed quality was the largest, while the environment substantially influenced the phenotypic traits of *X. sorbifolium* seeds, and rainfall was significantly negatively correlated with seed yield. Chai Chunshan et al. [24] divided the artificial cultivation population of *Xanthoceras sorbifolium* in Gansu Province into seven main factors, and the research results could provide reference for the selection of excellent cultivars of *Xanthoceras sorbifolium*. Detection of variation in plants based on phenotypic traits is a simple and easy method that can explain some components of the diversity. Therefore, from the perspective of the ornamental value of the genetic phenotypes of *Xanthoceras sorbifolium*, this study selected 33 cultivars of *Xanthoceras sorbifolium* to measure and analyze their phenotypic traits, such as leaves, flowers, and branches. Principal component analysis was used to comprehensively evaluate their phenotypic diversity, providing reference for screening ornamental *Xanthoceras sorbifolium* strains with different utilization values and laying a foundation for further improving the efficiency of the selection of ornamental *Xanthoceras sorbifolium* cultivars.

## 2. Results and Analysis

### 2.1. Phenotypic Diversity Analysis of Phenotypic Traits

#### 2.1.1. Diversity Analysis of Qualitative Traits

**Figure 1:**
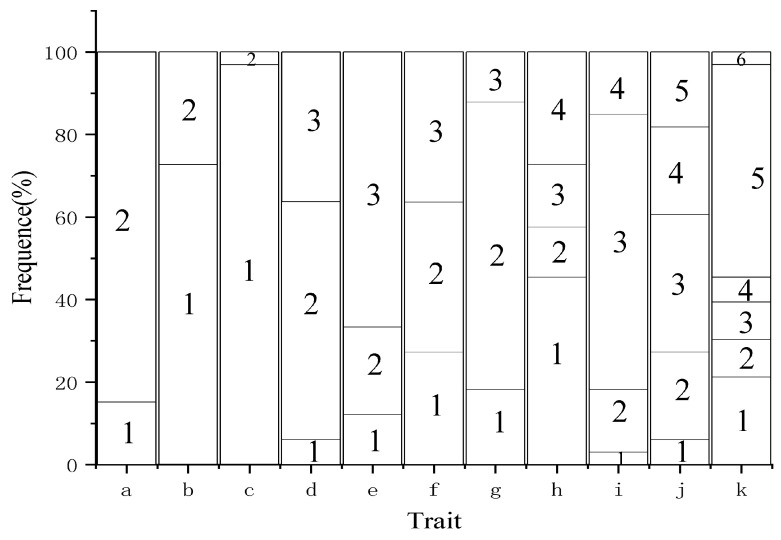
The variation distribution of qualitative traits of *Xanthoceras sorbifolium* cultivars: (a) the color of one-year branchlets; (b) presence of hair on one-year-old branchlets; (c) petal type; (d) leaf shape; (e) curl degree of leaflets; (f) the color of the lower part of the petals at S1; (g) the color of the upper part of the petals at S3; (h) the color of the upper part of the petals at S1; (i) the color of the lower part of the petals at S3; (j) the color of the lower part of the petals at S2; (k) the color of the upper part of the petals at S2).

The phenotypic diversity index of the qualitative traits ranged from 0.14 to 1.50 (average = 0.90) among the 33 cultivars analyzed (Table 1 and Table 2 and Figure 1). The lower and upper petal colors at S2 and S1 stages revealed a phenotypic diversity index of >1.00. Among them, the phenotypic diversity index of lower petal color in S2 was the highest (1.50), mainly showing orange cultivars with a frequency of 33.33%. Only QH4 and WH5 showed yellow cultivars in lower petals in the S2 stage. The phenotypic diversity index of the upper petal color in the S2 stage ranked second (1.38), mainly showing red cultivars with a frequency of 51.52%. In the S2 stage, only WH1 showed a purple line on the upper part of the petals. The phenotypic diversity index of upper petal color in the S1 stage was 1.25, with white, green-yellow, yellow-green, and yellow cultivars accounting for 45.45%, 12.12%, 15.15%, and 27.27% of the total frequency, respectively. The white had the highest frequency. The phenotypic diversity index of the lower petal color in the S1 stage was 1.09, showing yellow, yellow-green, and green-yellow colors, accounting for 27.27%, 36.36%, and 36.36%, respectively.

The phenotypic diversity index of the petal type was the lowest (0.14). The petal type could be divided into single and double petals, accounting for 96.97% and 3.03% of the total, respectively. Only WQC3 possessed double petals. The phenotypic diversity index for one-year-old branchlet colors was 0.43, with green and purplish and purplish red being the predominant strain, accounting for 84.85% of the total. The one-year-old branchlets of WQB3, WQB4, WQHQ, QH7, and WH3 were green and purplish. Of the total, 72.73% of one-year-old shoots were hairless, 27.27% were hairy, and their phenotypic diversity index was 0.59. The color of the upper portion of petals in S3 was classified into three types: white, red, and purpurine, with a phenotypic diversity index of 0.82, the red (69.70%) being the dominant one. The phenotypic diversity index for leaf shape and the degree of leaflet curling was 0.86. Leaf shapes were classified as lanceolate, ovate-lanceolate, and near ovate, accounting for 6.06%, 57.58%, and 36.36% of the total. The shapes were mainly ovate-lanceolate, and only QH7 and QH8 cultivars had lanceolate-shaped leaves. The leaflets were mainly curly (66.67%), but those of WQB2, WQB3, WQB4, and WQC3 cultivars were not curly. The phenotypic diversity index of the lower petal color at S3 was 0.95, with orange, orange-red, and red-purple cultivars accounting for 3.03%, 15.15%, 66.67%, and 15.15% of the total, respectively. The red cultivars appeared more frequently.

The coefficient of variation of the qualitative traits of ornamental *X. sorbifolium* cultivars ranged from 16.90% to 57.96%, and the average coefficient of variation was 32.39%, indicating a large degree of variation among the 11 qualitative traits. The variation degree from large to small was as follows: the color of the upper part of the petals at S1 > color of the upper part of the petals at S2 > color of the lower part of the petals at S1 > color of the lower part of the petals at S2 > presence of hair on one-year-old branchlets > color of the upper part of the petals at S3 > curl degree of leaflets > leaf shape > color of the lower part of the petals at S3 > color of one-year-old branchlets > petal type. Among these, the coefficient of variation of the upper petal color at S1 was the highest (57.96%), with the highest degree of variation; the coefficient of variation of petal type was the lowest (16.90%), with the lowest degree of variation.

#### 2.1.2. Diversity Analysis of Quantitative Traits

The phenotypic diversity index of quantitative traits ranged from 1.76 to 2.05, with an average of 1.92 (Table 3 and Table 4). The phenotypic diversity index of leaflet length was the highest (2.05), and that of bract length was the lowest (1.76). The other higher phenotypic diversity indexes were calyx width (2.01) and terminal inflorescence length (2.02). The coefficient of variation of the 17 quantitative traits ranged from 12.92% to 32.87%, with an average of 18.01%. Among these, the coefficient of variation for bract length (32.87%) was the largest, followed by lateral inflorescence length (22.00%). The coefficient of variation for leaflet length (12.92%) was the lowest, followed by petal length (13.12%). The degree of variation from large to small was as follows: bract length > lateral inflorescence length > pedicel length > bract width > number of flowers in the terminal inflorescence > flower width > number of flowers in the axillary inflorescence > length of the terminal inflorescence > petal width > leaf axis length > calyx width > large leaf length > flower length > calyx length > leaflet width > petal length > leaflet length.

The phenotypic diversity index of large leaf length was 1.98, and the coefficient of variation was 15.11%; the observation range was 11.47 (WQHD)–24.63 (WQC3) cm. The phenotypic diversity index of leaf axis length was 1.90, and the coefficient of variation was 16.07%; the observation range was 9.53 (WQHD)–22.60 (WQC3) cm. Other characteristics can be clearly seen in the table below.

### 2.2. PCA of Quantitative Traits

To determine whether the 17 quantitative traits were suitable for PCA, we performed the Kaiser–Meyer–Olkin (KMO) test and Bartlett’s spherical test using the quantitative traits of ornamental *X. sorbifolium* cultivars. The KMO was 0.665 (>0.6) and Sig was <0.05, suggesting that the quantitative traits supported PCA. Based on the principle that the characteristic value was greater than 1, the first five principal components (Table 5) were extracted, and the cumulative contribution rate was 79.82%, indicating that these principal components could represent the primary information of the phenotypes of ornamental *X. sorbifolium* cultivars. Among them, the contribution rate of the first principal component was the largest (31.70%), and the characteristic value was 5.39. Calyx length, petal length, petal width, flower length, and calyx width were the main indicators, with their characteristic vectors being 0.80, 0.73, 0.70, 0.70, and 0.66, respectively, which were the comprehensive reflection of flower size. The contribution rate of the second principal component was 24.51%, and the characteristic value was 4.17. Terminal inflorescence length, lateral inflorescence length, and flower width were the main indicators, and the characteristic vectors were 0.79, 0.78, and 0.68, respectively, which were mainly related to the size of the inflorescence. The contribution rate of the third principal component was 8.72%, and the eigenvalues were 1.48. The characters with higher absolute values of eigenvectors were mainly leaflet width (0.47) and bract width (0.42). The contribution rate of the fourth principal component was 8.51%, and the characteristic value was 1.45; bract length was the main index, and the characteristic vector was 0.66. The contribution rate of the fifth principal component was 6.38%, and the eigenvalues were 1.08. The character with a higher absolute value of eigenvectors was mainly leaflet length (0.46). Calyx length, terminal inflorescence length, lateral inflorescence length, petal length, petal width, flower length, and other characters contributed a lot to the quantitative traits of ornamental *X. sorbifolium* cultivars, which were related to flower morphology.

### 2.3. Comprehensive Evaluation of Phenotypic Traits

Based on the five principal component coefficients, the linear equations of *F*_1_, *F*_2_, *F*_3_, *F*_4_, and *F*_5_ were obtained, and the standardized data of 33 ornamental fruit lines for quantitative traits were substituted into the above five linear equations, and then the scores of each principal component of ornamental fruit lines were obtained (Table 6). Fi represents the score of the ith principal component, and *X*_1_, *X*_2_, *X*_3_ …… *X*_17_ represent the standardized data of quantitative traits of ornamental fruit lines.

*F*_1_= − 0.259 *X*_1_ − 0.251 *X*_2_ − 0.269 *X*_3_ − 0.193 *X*_4_ + 0.301 *X*_5_ + 0.237 *X*_6_ + 0.140 *X*_7_ + 0.316 *X*_8_ + 0.302 *X*_9_ + 0.346
*X*_10_ + 0.283 *X*_11_ + 0.215 *X*_12_ + 0.253 *X*_13_ − 0.073 *X*_14_ − 0.165 *X*_15_ − 0.074 *X*_16_ − 0.234 *X*_17,_ (1)

*F*_2_= 0.276 *X*_1_ + 0.270 *X*_2_ + 0.183 *X*_3_ + 0.147 *X*_4_ + 0.217 *X*_5_ + 0.262 *X*_6_ + 0.333 *X*_7_ + 0.218 *X*_8_ + 0.173 *X*_9_ + 0.170 *X*_10_ + 0.153 *X*_11_ − 0.070 *X*_12_ − 0.011 *X*_13_ + 0.388 *X*_14_ + 0.241 *X*_15_ + 0.382 *X*_16_ + 0.284 *X*_17_ (2)

*F*_3_= − 0.296 *X*_1_ − 0.259 *X*_2_ − 0.149 *X*_3_ + 0.383 *X*_4_ − 0.338 *X*_5_ − 0.324 *X*_6_ + 0.185 *X*_7_ + 0.031 *X*_8_ + 0.138 *X*_9_ − 0.166 *X*_10_ + 0.329 *X*_11_ − 0.103 *X*_12_ + 0.343 *X*_13_ + 0.019 *X*_14_ + 0.285 *X*_15_ + 0.049 *X*_16_ + 0.237 *X*_17_ (3)

*F*_4_= 0.248 *X*_1_ + 0.269 *X*_2_ + 0.116 *X*_3_ + 0.412 *X*_4_ − 0.030 *X*_5_ − 0.073 *X*_6_ − 0.269 *X*_7_ − 0.183 *X*_8_ + 0.063 *X*_9_ + 0.086 *X*_10_
+ 0.106 *X*_11_ + 0.545 *X*_12_ + 0.418 *X*_13_ − 0.013 *X*_14_ − 0.233 *X*_15_ + 0.077 *X*_16_ − 0.126 *X*_17_ (4)

*F*_5_= 0.123 *X*_1_ + 0.122 *X*_2_ + 0.445 *X*_3_ + 0.176 *X*_4_ − 0.170 *X*_5_ − 0.313 *X*_6_ + 0.220 *X*_7_ + 0.286 *X*_8_ + 0.215 *X*_9_ + 0.202 *X*_10_
+ 0.197 *X*_11_ − 0.250 *X*_12_ − 0.220 *X*_13_ − 0.170 *X*_14_ − 0.243 *X*_15_ − 0.284 *X*_16_ − 0.280 *X*_17_ (5)

Additionally, according to the five principal component coefficients and the contribution rate of each principal component, a comprehensive evaluation model of phenotypic diversity was constructed:

(6) $$F=(0.3171F_{1}+0.2451F_{2}+0.0872F_{3}+0.0851F_{4}+0.0638F_{5})/0.7982$$

The comprehensive scores of different *X. sorbifolium* cultivars were calculated using the model, and their phenotypic diversity was comprehensively evaluated. The F value of the 33 samples ranged from −2.79 to 1.93, in which the comprehensive scores of WQHC, QH8, WQHA, WQHQ, and WQC3 were <0, i.e., –1.15, –1.82, –2.24, –2.26, and –2.79, respectively, indicating a low phenotypic diversity. The comprehensive scores of WH10, QH5, WH5, WH8, WQHP, QH10, and WQHS were >1 and much higher than those of the other 16 cultivars, i.e., 1.93, 1.69, 1.40, 1.15, 1.12, 1.05, and 1.01, respectively, indicating a rich phenotypic diversity and that their phenotypic traits could effectively represent the diversity of phenotypic traits of multiple cultivars. Therefore, the selection, development, and utilization of fine idioplasm resources of *X. sorbifolium* should focus on these cultivars.

### 2.4. Cluster Analysis of Phenotypic Traits

**Figure 2:**
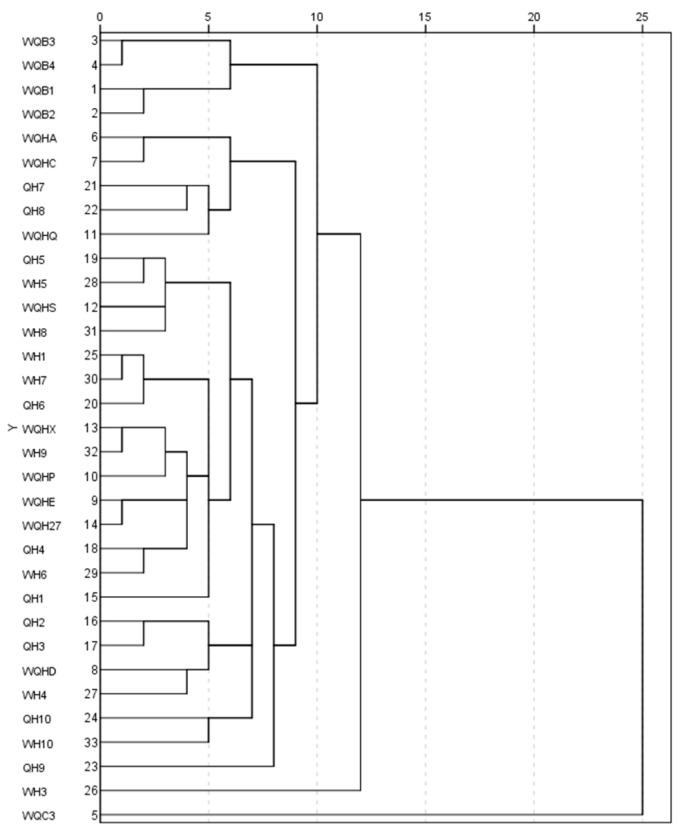
Clustering figure of ornamental *Xanthoceras sorbifolium* cultivars based on phenotypic characters.

The phenotypic traits of the 33 *X. sorbifolium* cultivars were analyzed using a systematic clustering method. The squared Euclidean distance was considered the genetic distance. When the genetic distance was 8.5, the tested *X. sorbifolium* cultivars were clustered into five categories (Figure 2).

The first category included four cultivars, which were mainly represented by the large leaf length, leaf axis length, leaflet length, leaflet width, flower width, petal length, calyx length, bract length, bract width, terminal inflorescence length, and lateral inflorescence length of each cultivar at a high level. The flower was a single-petal type. The color of the upper part of the petals at S1–S3 was white, and one-year-old branchlets were hairless, which could be used for breeding large white ornamental *X. sorbifolium* cultivars. The second category included five cultivars, mainly represented by large leaf length, leaf axis length, leaflet length, leaflet width, flower length, bract length, and calyx width belonging to the middle level. The flower width, petal length, petal width, calyx length, bract width, and number of flowers in the terminal inflorescence belonged to the lower level. The small leaves were curly, which could be used for breeding ornamental *X. sorbifolium* cultivars with small red flowers. The third category contained the most cultivars (22). The flower length, pedicel length, petal width, calyx length, calyx width, bract length, bract width, flower width, and petal length were all at a high level. The color of the annual branchlets was purplish red, which could be used for breeding cultivars with large red flowers. The fourth category contained only one cultivar, namely WH3, with small leaves, green and purplish red annual twigs, and large and dense flowers, which could be used for breeding large orange multi-flower cultivars. The fifth category also contained only one cultivar, namely WQC3. The flower length, pedicel length, flower width, petal length, petal width, calyx length, calyx width, bract length, and bract width were all at a lower level, which could be used for breeding double-petal cultivars.

## 3. Discussion 

The size and coefficient of variation of the plant genetic diversity index reflected the degree of variation and the level of genetic diversity of plant materials [25]. In this study, the quantitative traits were higher than the qualitative traits in phenotypic diversity, and the quantitative trait diversity was richer than the qualitative traits in *Xanthoceras sorbifolium*. This is consistent with the findings of Binbin Zhang [26], Qun Su et al. [27], and Shuangshuang Yi et al. [28]. Since all the experimental materials in this paper were taken from the *Xanthoceras Sorbifolium* Idioplasm Expo Park of Anqiu City, Shandong Province, the growth environment is consistent, which may not be able to fully reflect the morphological characteristics of *Xanthoceras Sorbifolium*, and further experiments can be conducted by selecting *Xanthoceras sorbifolium* cultivars with different growth environments because the qualitative traits are relatively stable, whereas quantitative traits are more susceptible to environmental conditions, genotype, idioplasm resource type, and other factors. In addition, the coefficient of variation of the qualitative traits of ornamental *X. sorbifolium* cultivars ranged from 16.90% to 57.96%, whereas that of quantitative traits ranged from 12.92% to 32.87%. The coefficient of variation greater than 10% may represent large differences between individuals [29,30]. Overall, the findings suggest that the phenotypic traits of the tested *X. sorbifolium* cultivars had relatively rich genetic variation.

The phenotypic diversity index of a plant phenotype represents the evenness of variation distribution, whereas the coefficient of variation represents the dispersion of variation [31]. Comparing the coefficient of variation and the phenotypic diversity index of the same character revealed that the level of the phenotypic diversity index was not consistent with that of the coefficient of variation. For example, the phenotypic diversity index of leaflet length was the highest; however, its coefficient of variation was the lowest. The coefficient of variation of the upper petal color at S1 was the highest, but the phenotypic diversity index was substantially low, similar to the findings of Liang et al. [32]. Among the phenotypic traits of the tested cultivars, the color of the upper part of the petals at S1, the color of the upper part of the petals at S2, the color of the lower part of the petals at S1, the color of the lower part of the petals at S2, presence of villi in one-year-old branchlets, and bract length had high coefficients of variation. The phenotypic diversity index of leaflet length, terminal inflorescence length, calyx width, and pedicel length was also high. The inconsistency between the levels of the phenotypic diversity index and the coefficient of variation indicates that the range of variation was large, and that the distribution of variation was uneven, which was also consistent with the findings of Tilman et al. [33].

Through PCA, 17 quantitative characters of 33 cultivars were extracted into five principal components, with a cumulative contribution rate of 79.82%, representing the vital information of quantitative traits of ornamental *X. sorbifolium* cultivars. A comprehensive evaluation model was built based on the PCA. The comprehensive score represented the richness in the diversity of plant phenotypic comprehensive traits. PCA has been widely used in the comprehensive evaluation of phenotypic traits of plants, such as roses [34] and water lilies [35]. The F value of the 33 samples ranged from −2.79 to 1.93, among which the comprehensive scores of WQHC, QH8, WQHA, WQHQ, and WQC3 were lower than 0, indicating that the phenotypic diversity of these cultivars was low; the comprehensive scores of WH10, QH5, WH5, WH8, WQHP, QH10, and WQHS were greater than 1, which was much higher than the other 16 cultivars, indicating a rich phenotypic diversity. Therefore, these cultivars must be considered for the screening, development, and utilization of fine idioplasm resources of *X. sorbifolium*.

Thirty-three ornamental *X. sorbifolium* cultivars were clustered into five categories by systematic clustering method. The four single petals cultivars with high levels of phenotypic traits, such as leaves and flowers, and with the white upper part of petals color from S1 to S3, were grouped into one group, which can be used for breeding ornamental *X. sorbifolium* cultivars with large white flowers. Different phenotypic indexes, such as leaves and flowers, were at the middle or lower level, and the five cultivars with red upper petal color at S3 were clustered into one group, which can be used for breeding red small-flowered ornamental *X. sorbifolium* cultivars. The 22 cultivars with high flower indexes and purple-red annual branchlets were clustered into one group, indicating that the genetic relationship between the 22 cultivars was relatively close, which can be used for breeding ornamental *X. sorbifolium* cultivars with large red flowers. WH3 had large and dense flowers, green and purplish red annual branchlets, and small leaves; WH3 was grouped into a single group, which can be used for breeding large, orange, and multi-flowered ornamental *X. sorbifolium* cultivars. The phenotypic indexes of WQC3 flowers were at a low level. The flower type was double, the leaflets were not curly, and the color of the upper part of the petals was white throughout the three stages. Therefore, it can be used for breeding double-petal ornamental *X. sorbifolium* cultivars. The cluster analysis results can be used for breeding ornamental *X. sorbifolium* cultivars with different utilization values. The findings of this study lay a foundation for further improving the efficiency of the breeding of ornamental *X. sorbifolium* cultivars.

## 4. Materials and Methods

### 4.1. Test Materials

**Figure 3:**
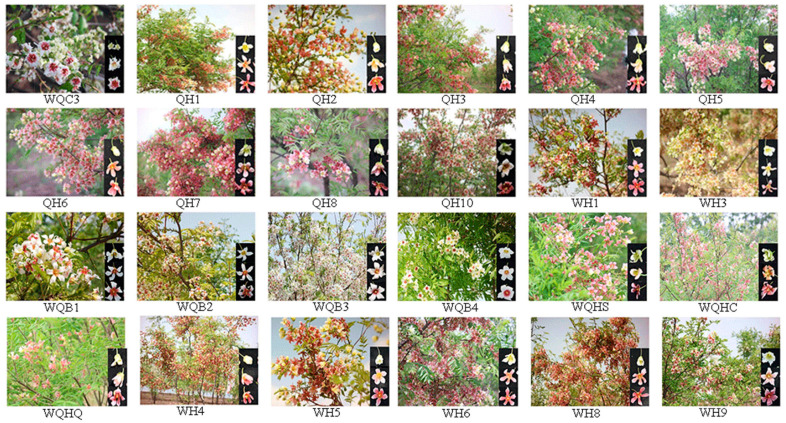
Some cultivars of ornamental *Xanthoceras sorbifolium*.

The test materials were stored in the *Xanthoceras Sorbifolium* Idioplasm Expo Park of Anqiu City, Shandong Province, located at the northeast edge of the central mountain area of Shandong Province between 118°44′ E–119°27′ E and 36°05′ N–36°38′ N. The terrain was high in the southwest and low in the northeast, with a mild temperate continental monsoon climate and four distinct seasons. *Xanthoceras Sorbifolium* Idioplasm Expo Park of Anqiu City covered an area of 400 mu, with 370,000 *X. sorbifolium* trees. It is the largest and most complete *Xanthoceras Sorbifolium* idioplasm resources conservation gene bank in China. In this experiment, 33 cultivars with beautiful trunk shapes, consistent growth, and robust basal growth were selected as the test materials to measure and record the phenotypic traits, such as branches, leaves, and flowers. A completely randomized group design was used, with a group of 10 plants and 3 replications, and all germplasms were subjected to uniform field management measures. The flowering period of *X. sorbifolium* could be divided into S1 (blooming flower, the lower part of the petal is green to yellow), S2 (fully opened flower, the petal began to change color, the lower part of the petal was yellow to red), and S3 (the petal color changed completely, the lower part of the petal was orange or red). A total of 3 to 10 plants of each species were collected, and at least 50 flowers were collected from each plant. The names of the test materials are listed in Table 7. Some cultivars of ornamental *Xanthoceras sorbifolium* are shown in Figure 3.

### 4.2. Test Methods

In total, 28 morphological indicators were investigated. This study referred to the standards of Zhou Shuai [36], and 11 qualitative traits were investigated through the observation and comparison method, including the color of one-year branchlets, presence of hair on one-year-old branchlets, petal type, leaf shape, degree of leaf curl, the color of the lower and upper part of the petals in all sections (S1, S2, and S3). The quantitative traits were measured using specific methods with 17 indicators, including length of large leaf, length of leaf axis, length of leaflet, width of leaflet, length of flower, length of pedicel, width of flower, length of petal, width of petal, length of calyx, width of calyx, length of bract, width of bract, length of terminal inflorescence, number of flowers in terminal inflorescence, length of lateral inflorescence, and number of flowers in lateral inflorescence. The observation of color characteristics was based on the colorimetric card published by the Royal Horticultural Association of the United Kingdom [37]. The color test used a white background plate. The tools required for collecting the observation data mainly included a colorimetric card, tape measure, scale, and vernier caliper.

The phenotypic diversity index, coefficient of variation, and the average coefficient of variation of phenotypic characters of *X. sorbifolium* were analyzed using Excel 2016 software. Qualitative traits were graded on a 1–6 scale and assigned values (Table 8). Based on the mean (X) and standard deviation (δ), quantitative traits were classified into 10 levels, with Grade 1 < X − 2δ and Grade 10 ≥ X + 2δ, and each stage differed by 0.5 δ. The phenotypic diversity of each trait was evaluated using the Shannon–Weaver diversity index: *H′* = $-\sum \left(P_{i}\right)$($\ln P_{i}$), where *P_i_* denotes the frequency of occurrence of the i-th variant type [38]. SPSS 26 software was used to conduct principal component analysis (PCA) and cluster analysis of the quantitative characters of the tested materials. In the PCA, the principal component was extracted based on the characteristic value >1, and a comprehensive evaluation model was constructed. The comprehensive score of different *X. sorbifolium* varietiescultivars was calculated using the model. A systematic cluster analysis was conducted on 33 ornamental *X. sorbifolium* cultivars.

## 5. Conclusions

In conclusion, the phenotypic diversity of the cultivars in this experiment was higher for quantitative traits than for qualitative traits, and the diversity of quantitative traits was richer than that of qualitative traits. For the cultivars with high phenotypic diversity, we can focus on the screening and development of ornamental *Xanthoceras Sorbifolium* germplasm resources in the future. The systematic clustering of 33 ornamental *Xanthoceras Sorbifolium* cultivars into five categories can provide reference for the selection and breeding of ornamental *Xanthoceras Sorbifolium* with different utilization values. However, due to the limitation of sampling sites and sampling time, fewer white and orange *Xanthoceras sorbifolium* cultivars were used in the experiment, so the number of various species of *Xanthoceras sorbifolium* should be increased in future studies to enrich the research results. As a flowering species with high ornamental value, the flowers of Corolla are large and numerous with brilliant colors, but the flowers of the plant lack certain specific colors, such as pure purple varieties and highly saturated orange cultivars. In future research and cultivars selection work, breeding such cultivars will be beneficial to enrich the flower color of *Xanthoceras sorbifolium*.

## Figures and Tables

**Table 1:** Analysis of diversity of qualitative characters of ornamental *Xanthoceras sorbifolium* cultivars.

Traits	Phenotypic Diversity Index H′	Coefficient of Variation/%
The color of one-year branchlets	0.43	19.7
Presence of hair on one-year-old branchlets	0.59	35.54
Petal type	0.14	16.9
Leaf shape	0.86	25.42
Curl degree of leaflets	0.86	27.94
The color of the lower part of the petals at S1	1.09	38.49
The color of the upper part of the petals at S3	0.82	28.65
The color of the upper part of the petals at S1	1.25	57.96
The color of the lower part of the petals at S3	0.95	22.4
The color of the lower part of the petals at S2	1.50	36.19
The color of the upper part of the petals at S2	1.38	47.07

**Table 2:** Characteristics of qualitative characters of ornamental *Xanthoceras sorbifolium* cultivars.

Cultivars Name	The Color of One-Year Branchlets		Presence of Hair on One-Year-Old Branchlets	Petal Type	Leaf Shape	Curl Degree of Leaflets
WQB1	Purplish red		Were hairless	Single petal	Ovate-lanceolate	Micro curly
WQB2	Purplish red		Were hairless	Single petal	Ovate-lanceolate	Not curly
WQB3	With green and purplish red		Were hairless	Single petal	Near ovate	Not curly
WQB4	With green and purplish red		Were hairless	Single petal	Near ovate	Not curly
WQC3	Purplish red		Were hairless	Double petals	Near ovate	Not curly
WQHA	Purplish red		Were hairless	Single petal	Ovate-lanceolate	Mainly curly
WQHC	Purplish red		Were hairless	Single petal	Ovate-lanceolate	Micro curly
WQHD	Purplish red		Were hairless	Single petal	Ovate-lanceolate	Mainly curly
WQHE	Purplish red		Were hairless	Single petal	Ovate-lanceolate	Micro curly
WQHP	Purplish red		Were hairless	Single petal	Ovate-lanceolate	Micro curly
WQHQ	With green and purplish red		Were hairy	Single petal	Ovate-lanceolate	Mainly curly
WQHS	Purplish red		Were hairless	Single petal	Ovate-lanceolate	Mainly curly
WQHX	Purplish red		Were hairless	Single petal	Ovate-lanceolate	Micro curly
WQH27	Purplish red		Were hairless	Single petal	Ovate-lanceolate	Micro curly
QH1	Purplish red		Were hairy	Single petal	Near ovate	Mainly curly
QH2	Purplish red		Were hairless	Single petal	Near ovate	Mainly curly
QH3	Purplish red		Were hairless	Single petal	Ovate-lanceolate	Mainly curly
QH4	Purplish red		Were hairless	Single Petal	Ovate-lanceolate	Mainly curly
QH5	Purplish red		Were hairy	Single Petal	Near ovate	Mainly curly
QH6	Purplish red		Were hairless	Single Petal	Near ovate	Mainly curly
QH7	With green and purplish red		Were hairy	Single Petal	Lanceolate	Mainly curly
QH8	Purplish red		Were hairy	Single Petal	Lanceolate	Mainly curly
QH9	Purplish red		Were hairless	Single Petal	Ovate-lanceolate	Mainly curly
QH10	Purplish red		Were hairy	Single petal	Ovate-lanceolate	Mainly curly
WH1	Purplish red		Were hairless	Single petal	Near ovate	Mainly curly
WH3	With green and purplish red		Were hairless	Single petal	Near ovate	Mainly curly
WH4	Purplish red		Were hairy	Single petal	Near ovate	Mainly curly
WH5	Purplish red		Were hairy	Single petal	Near ovate	Micro curly
WH6	Purplish red		Were hairy	Single petal	Ovate-lanceolate	Mainly curly
WH7	Purplish red		Were hairless	Single petal	Ovate-lanceolate	Mainly curly
WH8	Purplish red		Were hairless	Single petal	Near ovate	Mainly curly
WH9	Purplish red		Were hairless	Single petal	Ovate-lanceolate	Mainly curly
WH10	Purplish red		Were hairless	Single petal	Ovate-lanceolate	Mainly curly
**Cultivars Name**	**The Color of the Lower Part of the Petals at S1**	**The Color of the Upper Part of the Petals at S3**	**The Color of the Upper Part of the Petals at S1**	**The Color of the Lower Part of the Petals at S3**	**The Color of the Lower Part of the Petals at S2**	**The Color of the Upper Part of the Petals at S2**
WQB1	White group	Yellow group	White group	Orange group	White group	Red group
WQB2	White group	Yellow group	White group	Orange group	White group	Red-violet group
WQB3	White group	Green-yellow group	White group	Orange group	White group	Red group
WQB4	White group	Green-yellow group	White group	Red group	White group	Red group
WQC3	White group	Yellow group	White group	Red group	White group	Red group
WQHA	Yellow group	Yellow group	Yellow-orange group	Orange-red group	Red group	Orange-red group
WQHC	Yellow-green group	Yellow-green group	Yellow-orange group	Orange-red group	Red group	Orange-red group
WQHD	Yellow group	Yellow-green group	Orange group	Orange group	Red group	Red group
WQHE	Yellow group	Yellow group	Orange group	Orange-red group	Red group	Red group
WQHP	Yellow group	Yellow group	Yellow group	Orange group	Red group	Red group
WQHQ	Yellow group	Green-yellow group	Red group	Yellow-orange group	Red group	Red group
WQHS	Yellow-green group	Yellow-green group	Red group	Orange group	Red group	Orange-red group
WQHX	Yellow group	Green-yellow group	Red group	Red group	Red group	Red group
WQH27	Yellow group	Green-yellow group	Red group	Orange-red group	Red group	Red group
QH1	Green-yellow group	Green-yellow group	Yellow group	Yellow-orange group	Red group	Red group
QH2	White group	Yellow group	Red group	Orange group	Red group	Orange-red group
QH3	Yellow-green group	Yellow-green group	Red group	Yellow-orange group	Red group	Red group
QH4	Yellow-green group	Green-yellow group	Red group	Yellow group	Red group	Red group
QH5	White group	Yellow-green group	Red group	Yellow-orange group	Red-violet group	Red-violet group
QH6	Green-yellow group	Green-yellow group	Yellow-orange group	Orange group	Red-violet group	Red-violet group
QH7	White group	Yellow-green group	Red group	Red group	Red group	Red group
QH8	White group	Green-yellow group	Red group	Red group	Red group	Red group
QH9	White group	Green-yellow group	Red group	Red group	Red group	Red group
QH10	White group	Green-yellow group	White group	Yellow-orange group	Red group	Red group
WH1	White group	Yellow-green group	Violet group	Orange-red group	Red-violet group	Red-violet group
WH3	White group	Yellow-green group	Red group	Orange-red group	Red group	Orange group
WH4	Yellow group	Yellow group	Yellow group	Orange group	Red group	Red group
WH5	Green-yellow group	Yellow-green group	Red group	Yellow group	Red group	Red group
WH6	Green-yellow group	Yellow-green group	Red group	Yellow-orange group	Red group	Red group
WH7	White group	Yellow group	Red group	Orange group	Red-violet group	Red-violet group
WH8	Yellow-green group	Yellow-green group	Red group	Yellow-orange group	Red group	Red group
WH9	Yellow group	Yellow-green group	Red group	Orange-red group	Red group	Red group
WH10	White group	Green-yellow group	White group	Orange group	White group	Orange-red group

**Table 3:** Analysis of diversity of quantitative characters of ornamental *Xanthoceras sorbifolium* cultivars.

Traits	Average	Standard Deviation	Max	Min	Range	Phenotypic Diversity Indexh’	Coefficient of Variation/%
Large leaf length	18.03	2.72	24.63	11.47	13.17	1.98	15.11
Leaf axis length	15.24	2.45	22.60	9.53	13.07	1.90	16.07
Leaflet length	4.44	0.57	5.63	3.27	2.37	2.05	12.92
Leaflet width	1.47	0.20	1.93	1.07	0.87	1.96	13.44
Length of the terminal inflorescence	21.62	4.01	30.07	12.80	17.27	2.02	18.54
Number of flowers in the terminal inflorescence	29.83	5.85	51.33	20.00	31.33	1.84	19.61
Lateral inflorescence length	16.34	3.60	26.50	9.77	16.73	1.96	22.00
Number of flowers in the axillary inflorescence	24.36	4.72	35.00	14.67	20.33	1.86	19.35
Flower length	3.71	0.56	4.70	2.37	2.33	1.96	14.96
Pedicel length	2.01	0.41	2.88	1.14	1.73	2.00	20.64
Flower width	2.31	0.45	3.06	1.38	1.68	1.84	19.43
Petal length	1.88	0.25	2.31	1.20	1.11	1.79	13.12
Petal width	0.79	0.14	1.04	0.43	0.61	1.97	17.64
Calyx length	0.80	0.12	1.16	0.56	0.60	1.89	14.61
Calyx width	0.34	0.05	0.46	0.24	0.22	2.01	15.71
Bract length	0.54	0.18	1.27	0.34	0.92	1.76	32.87
Bract width	0.30	0.06	0.46	0.22	0.23	1.89	20.09

**Table 4:** Characteristics of quantitative characters of ornamental *Xanthoceras sorbifolium* cultivars.

Cultivars Name	Large Leaf Length/cm	Leaf Axis Length/cm	Leaflet Length/cm	Leaflet Width/cm	Terminal Inflorescence Length/cm	Number of Flowers in the Terminal Inflorescence
WQB1	20.83 ± 0.81	18.1 ± 1.3	4.33 ± 0.23	1.5 ± 0.17	30.07 ± 2	31.33 ± 0.58
WQB2	17.4 ± 1.21	14.23 ± 0.91	5.13 ± 0.25	1.2 ± 0.1	21 ± 2.14	28.33 ± 1.53
WQB3	19.63 ± 2.42	17.57 ± 2.93	4.83 ± 0.4	1.57 ± 0.25	26.9 ± 1.44	35.67 ± 6.35
WQB4	22.9 ± 1.8	17.9 ± 2.39	5.63 ± 0.75	1.6 ± 0.1	30 ± 2.65	34.33 ± 4.73
WQC3	24.63 ± 0.45	22.6 ± 0.76	5.43 ± 0.57	1.77 ± 0.15	23.67 ± 1.88	40.33 ± 4.04
WQHA	16.5 ± 0.3	13.33 ± 0.91	3.53 ± 0.23	1.3 ± 0	19.37 ± 2.72	28 ± 1
WQHC	16.43 ± 1.5	13.87 ± 0.65	5.07 ± 0.15	1.77 ± 0.06	18.57 ± 1.1	27.33 ± 4.16
WQHD	11.47 ± 1.8	9.53 ± 1.27	3.93 ± 0.85	1.3 ± 0.1	19.4 ± 1.15	23 ± 5
WQHE	20.33 ± 1	17.67 ± 1.17	5.27 ± 1.34	1.33 ± 0.15	23.77 ± 3.89	29 ± 4.58
WQHP	19.8 ± 0.5	16.27 ± 1.06	4.3 ± 0.46	1.6 ± 0.36	25.4 ± 1.65	36.33 ± 3.22
WQHQ	17.8 ± 1.35	14.73 ± 1.27	4.47 ± 0.23	1.27 ± 0.15	20.37 ± 0.98	32.33 ± 2.52
WQHS	17.3 ± 1.01	14.27 ± 1.05	4.27 ± 0.15	1.27 ± 0.06	25.43 ± 3.49	25.33 ± 4.16
WQHX	19.67 ± 1.52	16.4 ± 0.98	4.03 ± 0.23	1.47 ± 0.21	21.57 ± 2.56	31.67 ± 2.52
WQH27	22.13 ± 7.09	16.7 ± 2.08	5.1 ± 0.52	1.4 ± 0.17	18.17 ± 1.65	23.67 ± 4.04
QH1	19.1 ± 1.04	16.1 ± 0.95	4.67 ± 0.4	1.73 ± 0.21	27.8 ± 3.06	33.67 ± 2.52
QH2	15.3 ± 2.59	13.13 ± 1.8	3.7 ± 0.56	1.43 ± 0.25	20.17 ± 0.61	29.33 ± 12.66
QH3	15.47 ± 1.59	13.33 ± 1.72	3.63 ± 0.25	1.4 ± 0	19.5 ± 1.18	27.33 ± 1.16
QH4	21.2 ± 1.77	18.37 ± 1.62	5.1 ± 0.35	1.5 ± 0.1	21.03 ± 1.91	28 ± 3.61
QH5	18.3 ± 1.05	15.57 ± 1.06	3.97 ± 0.29	1.17 ± 0.06	24 ± 3.28	32 ± 4.36
QH6	19.4 ± 1.21	16 ± 1.13	4.57 ± 0.4	1.67 ± 0.25	23 ± 3.5	25 ± 6.56
QH7	18.33 ± 1.79	15.37 ± 1.24	4.83 ± 0.32	1.53 ± 0.21	19.13 ± 1.61	31.67 ± 2.52
QH8	15.5 ± 2.07	13 ± 1.01	4.7 ± 1.15	1.5 ± 0.53	12.8 ± 1.95	32.67 ± 5.51
QH9	18.93 ± 0.55	16.13 ± 0.46	4.2 ± 0.4	1.53 ± 0.23	16.83 ± 1.89	27 ± 4.58
QH10	15.53 ± 2.22	12.37 ± 2.18	4.13 ± 0.58	1.57 ± 0.25	20.6 ± 1.56	22.67 ± 1.16
WH1	15.2 ± 1.13	12.33 ± 0.97	4.17 ± 0.6	1.53 ± 0.21	18.7 ± 4.07	30 ± 2.65
WH3	14.83 ± 1.83	12.43 ± 1.47	3.97 ± 0.51	1.43 ± 0.25	25.1 ± 4.23	51.33 ± 5.69
WH4	14.4 ± 1.66	12.53 ± 1.14	3.27 ± 0.15	1.07 ± 0.15	17.53 ± 1.03	20 ± 2.65
WH5	18.23 ± 1.1	16.13 ± 0.7	4.2 ± 0.1	1.53 ± 0.12	20.57 ± 0.86	30.33 ± 0.58
WH6	18.77 ± 2.08	16.57 ± 1.8	4.6 ± 0.1	1.73 ± 0.06	18.37 ± 1.01	29 ± 5.29
WH7	17.57 ± 1.01	14.63 ± 1.12	4.67 ± 0.61	1.57 ± 0.15	22.1 ± 3.83	27 ± 2.65
WH8	16.43 ± 0.29	14.03 ± 0.12	3.9 ± 0.3	1.3 ± 0.1	14.53 ± 0.4	26.33 ± 1.53
WH9	19.33 ± 1.12	16.73 ± 1.59	4.7 ± 0.17	1.93 ± 0.15	25 ± 2	31.67 ± 5.77
WH10	15.77 ± 1.06	14.2 ± 1.06	4.37 ± 0.74	1.27 ± 0.12	23.17 ± 1	22.67 ± 0.58
**Cultivars Name**	**Lateral Inflorescence** **/cm**	**Number of Flowers in the Axillary**	**Flower Length** **/cm**	**Pedicel Length/cm**	**Flower Width** **/cm**	**Petal Length** **/cm**
WQB1	15.7 ± 0.52	24.33 ± 3.06	3.64 ± 0.45	2.00 ± 0.26	2.88 ± 0.31	2.08 ± 0.20
WQB2	15.27 ± 2.65	23.67 ± 2.08	3.59 ± 0.55	1.88 ± 0.16	2.49 ± 0.3	2.12 ± 0.22
WQB3	21.77 ± 1.99	28.67 ± 1.16	3.79 ± 0.45	2.12 ± 0.19	2.83 ± 0.32	2.18 ± 0.16
WQB4	26.5 ± 1.50	32.33 ± 2.52	3.72 ± 0.33	2.21 ± 0.24	2.48 ± 0.39	1.79 ± 0.34
WQC3	17.8 ± 1.05	34.33 ± 0.58	2.37 ± 0.30	1.21 ± 0.16	1.56 ± 0.38	1.2 ± 0.30
WQHA	15.9 ± 2.12	27.33 ± 2.08	3.1 ± 0.41	1.62 ± 0.28	1.86 ± 0.27	1.39 ± 0.18
WQHC	14.87 ± 2.66	23 ± 1.00	3.19 ± 0.55	1.79 ± 0.43	2.22 ± 0.27	1.62 ± 0.18
WQHD	14.33 ± 1.29	16 ± 1.00	3.02 ± 0.67	1.72 ± 0.45	2.53 ± 0.17	2.12 ± 0.15
WQHE	18.9 ± 1.37	24.33 ± 4.73	4.1 ± 0.57	2.49 ± 0.76	2.39 ± 0.71	1.82 ± 0.32
WQHP	18.03 ± 2.92	35 ± 4.58	4.26 ± 0.49	2.56 ± 0.52	2.66 ± 0.59	2 ± 0.26
WQHQ	11.97 ± 1.63	28 ± 3.61	2.77 ± 0.44	1.14 ± 0.33	1.72 ± 0.45	1.66 ± 0.31
WQHS	20 ± 1.61	22.33 ± 9.02	4.57 ± 0.64	2.54 ± 0.4	2.66 ± 0.54	2.1 ± 0.17
WQHX	18.83 ± 0.9	25 ± 3.61	3.4 ± 0.32	1.79 ± 0.38	2.5 ± 0.82	1.8 ± 0.26
WQH27	12.97 ± 1.81	17.67 ± 1.16	3.97 ± 0.38	2.19 ± 0.24	1.92 ± 0.76	1.73 ± 0.26
QH1	21.03 ± 0.64	26.67 ± 1.16	3.49 ± 0.44	2.31 ± 0.19	2.52 ± 0.51	2 ± 0.18
QH2	18.37 ± 1.67	21.33 ± 6.66	4.48 ± 0.3	2.68 ± 0.36	1.53 ± 0.18	1.74 ± 0.07
QH3	12.03 ± 1.53	20.67 ± 3.22	3.81 ± 0.41	2.13 ± 0.26	1.89 ± 0.44	1.82 ± 0.14
QH4	15.43 ± 1.58	23.33 ± 0.58	3.37 ± 0.26	1.66 ± 0.34	1.86 ± 0.6	1.74 ± 0.13
QH5	19.73 ± 1.59	26.67 ± 3.06	4.7 ± 0.55	2.88 ± 0.43	2.91 ± 0.57	2.08 ± 0.17
QH6	16.43 ± 1.57	24.33 ± 3.51	3.89 ± 0.43	2.06 ± 0.19	2.11 ± 0.49	1.72 ± 0.1
QH7	15.73 ± 0.67	28 ± 4.36	3.71 ± 0.31	1.98 ± 0.3	2.56 ± 0.77	1.88 ± 0.24
QH8	9.77 ± 0.42	22.67 ± 3.06	3.01 ± 0.42	1.47 ± 0.27	1.74 ± 0.56	1.59 ± 0.2
QH9	12.6 ± 0.53	14.67 ± 4.93	4 ± 0.29	2.04 ± 0.24	1.99 ± 0.55	1.83 ± 0.05
QH10	16.5 ± 0.76	23.67 ± 1.16	3.22 ± 0.33	1.53 ± 0.19	2.32 ± 0.37	1.93 ± 0.1
WH1	17.03 ± 2.29	25 ± 3.46	3.3 ± 0.19	1.78 ± 0.33	2.5 ± 0.42	1.8 ± 0.12
WH3	15.53 ± 3.26	26.67 ± 6.81	4.26 ± 0.42	2.27 ± 0.23	2.87 ± 0.67	2.13 ± 0.19
WH4	11.4 ± 1.05	17.67 ± 1.53	3.61 ± 0.37	1.81 ± 0.38	1.38 ± 0.26	1.68 ± 0.16
WH5	18.43 ± 1.03	25.67 ± 2.08	4.27 ± 0.35	2.24 ± 0.17	2.69 ± 0.68	2.24 ± 0.1
WH6	12.4 ± 1.05	19.67 ± 2.31	3.84 ± 0.38	1.83 ± 0.2	2.77 ± 0.66	2.00 ± 0.39
WH7	19.5 ± 1.05	26.33 ± 0.58	4.09 ± 0.53	2.4 ± 0.19	2.47 ± 0.54	1.78 ± 0.26
WH8	12.03 ± 1.58	23.67 ± 2.52	4.42 ± 0.55	2.42 ± 0.18	3.06 ± 0.98	2.21 ± 0.15
WH9	19.67 ± 2.08	27.33 ± 5.03	3.21 ± 0.29	1.43 ± 0.23	2.51 ± 0.82	1.86 ± 0.27
WH10	12.9 ± 1.22	18 ± 1.73	4.33 ± 0.34	2.03 ± 0.29	2.00 ± 0.4	2.31 ± 0.14
**Cultivars Nname**	**Petal Width** **/cm**	**Calyx Length** **/cm**	**Calyx Width** **/cm**	**Bract Length** **/cm**		**Bract Width** **/cm**
WQB1	0.72 ± 0.11	0.79 ± 0.09	0.3 ± 0.05	0.43 ± 0.07		0.28 ± 0.07
WQB2	0.58 ± 0.08	0.81 ± 0.08	0.28 ± 0.04	0.41 ± 0.08		0.22 ± 0.04
WQB3	0.89 ± 0.08	0.82 ± 0.08	0.32 ± 0.04	0.39 ± 0.03		0.28 ± 0.04
WQB4	0.91 ± 0.08	0.82 ± 0.1	0.29 ± 0.03	0.49 ± 0.15		0.28 ± 0.07
WQC3	0.43 ± 0.07	0.56 ± 0.12	0.28 ± 0.04	0.38 ± 0.1		0.23 ± 0.09
WQHA	0.61 ± 0.11	0.58 ± 0.04	0.24 ± 0.05	0.34 ± 0.07		0.22 ± 0.08
WQHC	0.77 ± 0.1	0.63 ± 0.07	0.29 ± 0.03	0.38 ± 0.08		0.26 ± 0.05
WQHD	0.89 ± 0.13	0.81 ± 0.16	0.37 ± 0.07	0.49 ± 0.08		0.31 ± 0.06
WQHE	0.77 ± 0.1	0.77 ± 0.12	0.36 ± 0.05	0.4 ± 0		0.24 ± 0.05
WQHP	0.84 ± 0.1	0.82 ± 0.08	0.4 ± 0	0.54 ± 0.1		0.36 ± 0.07
WQHQ	0.54 ± 0.1	0.66 ± 0.07	0.26 ± 0.05	0.43 ± 0.19		0.23 ± 0.07
WQHS	0.89 ± 0.14	0.94 ± 0.1	0.32 ± 0.04	0.67 ± 0.29		0.26 ± 0.05
WQHX	0.83 ± 0.05	0.81 ± 0.13	0.39 ± 0.06	0.53 ± 0.09		0.29 ± 0.06
WQH27	0.84 ± 0.09	0.83 ± 0.07	0.34 ± 0.05	0.36 ± 0.09		0.22 ± 0.04
QH1	0.59 ± 0.12	0.82 ± 0.04	0.39 ± 0.03	0.51 ± 0.15		0.28 ± 0.05
QH2	0.71 ± 0.13	0.82 ± 0.15	0.33 ± 0.05	0.83 ± 0.26		0.41 ± 0.08
QH3	0.83 ± 0.15	0.93 ± 0.19	0.38 ± 0.04	0.72 ± 0.19		0.39 ± 0.09
QH4	0.72 ± 0.1	0.77 ± 0.12	0.3 ± 0	0.49 ± 0.08		0.3 ± 0.09
QH5	0.99 ± 0.13	1 ± 0.11	0.37 ± 0.05	0.63 ± 0.17		0.32 ± 0.07
QH6	0.64 ± 0.07	0.79 ± 0.2	0.3 ± 0.05	0.58 ± 0.12		0.24 ± 0.05
QH7	0.76 ± 0.09	0.81 ± 0.08	0.39 ± 0.06	0.44 ± 0.26		0.28 ± 0.13
QH8	0.72 ± 0.1	0.7 ± 0.09	0.28 ± 0.04	0.48 ± 0.2		0.22 ± 0.04
QH9	0.73 ± 0.11	0.77 ± 0.09	0.32 ± 0.04	1.27 ± 2		0.29 ± 0.06
QH10	0.97 ± 0.11	0.88 ± 0.08	0.4 ± 0.07	0.78 ± 0.23		0.46 ± 0.11
WH1	0.78 ± 0.08	0.71 ± 0.09	0.36 ± 0.05	0.57 ± 0.15		0.41 ± 0.12
WH3	0.84 ± 0.09	0.78 ± 0.08	0.42 ± 0.04	0.41 ± 0.03		0.26 ± 0.05
WH4	0.77 ± 0.1	0.73 ± 0.07	0.33 ± 0.05	0.52 ± 0.07		0.32 ± 0.04
WH5	1.04 ± 0.12	0.92 ± 0.11	0.38 ± 0.07	0.58 ± 0.08		0.29 ± 0.08
WH6	0.74 ± 0.1	0.84 ± 0.12	0.34 ± 0.05	0.59 ± 0.15		0.36 ± 0.15
WH7	0.76 ± 0.07	0.73 ± 0.07	0.31 ± 0.06	0.58 ± 0.14		0.32 ± 0.07
WH8	0.86 ± 0.15	0.92 ± 0.12	0.41 ± 0.06	0.47 ± 0.1		0.31 ± 0.06
WH9	0.96 ± 0.11	0.73 ± 0.05	0.43 ± 0.08	0.5 ± 0.11		0.31 ± 0.06
WH10	0.98 ± 0.15	1.16 ± 0.22	0.46 ± 0.07	0.71 ± 0.15		0.36 ± 0.05

**Table 5:** Principal component analysis of quantitative characters of ornamental *Xanthoceras sorbifolium* cultivars.

Traits	Principal				
	1	2	3	4	5
Large leaf length	−0.60	0.56	−0.36	0.30	0.13
Leaf axis length	−0.58	0.55	−0.32	0.32	0.13
Leaflet length	−0.63	0.37	−0.18	0.14	0.46
Mainly leaflet width	−0.45	0.30	0.47	0.50	0.18
Flower length	0.70	0.44	−0.41	−0.04	−0.18
Pedicel length	0.55	0.53	−0.39	−0.09	−0.33
Flower width	0.33	0.68	0.23	−0.32	0.23
Petal length	0.73	0.45	0.04	−0.22	0.30
Petal width	0.70	0.35	0.17	0.08	0.22
Calyx length	0.80	0.35	−0.20	0.10	0.21
Calyx width	0.66	0.31	0.40	0.13	0.21
Bract length	0.50	−0.14	−0.13	0.66	−0.26
Bract width	0.59	−0.02	0.42	0.50	−0.23
Terminal inflorescence length	−0.17	0.79	0.02	−0.02	−0.18
Number of flowers in the terminal inflorescence	−0.38	0.49	0.35	−0.28	−0.25
Lateral inflorescence length	−0.17	0.78	0.06	0.09	−0.30
Number of flowers in the axillary inflorescence	−0.54	0.58	0.29	−0.15	−0.29
Characteristic value	5.39	4.17	1.48	1.45	1.08
Contribution rate/%	31.71	24.51	8.72	8.51	6.38
Cumulative contribution rate/%	31.71	56.22	64.94	73.45	79.82

**Table 6:** Comprehensive evaluation about quantitative characters of ornamental *Xanthoceras sorbifolium* cultivars.

Cultivars Name	Principal Component Score					Comprehensive Scores/F	Ranking
	F1	F2	F3	F4	F5		
WH10	4.46	0.00	−0.56	0.94	1.51	1.93	1
QH5	2.94	2.76	−1.44	−0.68	−1.21	1.69	2
WH5	1.97	1.86	0.08	−0.06	0.67	1.40	3
WH8	3.16	−0.06	−0.17	−1.34	0.96	1.15	4
WQHP	0.46	2.97	0.98	0.13	−1.22	1.12	5
QH10	2.24	−1.08	2.32	2.04	0.24	1.05	6
WQHS	2.20	1.42	−1.74	−0.65	−0.48	1.01	7
WH3	1.19	1.79	2.02	−2.81	−0.95	0.86	8
WQB3	−0.69	3.11	0.29	−0.72	0.32	0.66	9
WH9	−1.13	1.59	2.54	1.38	1.20	0.56	10
QH3	2.47	−1.76	0.32	1.00	−0.71	0.53	11
WH6	0.48	−0.22	0.36	1.10	1.40	0.39	12
QH2	2.07	−1.11	−0.53	1.49	−2.96	0.35	13
QH1	−0.95	2.16	0.60	0.17	−0.33	0.35	14
WQHD	2.55	−2.63	1.46	−1.41	1.02	0.30	15
WQB4	−2.34	4.05	−0.51	0.63	−0.43	0.29	16
WQHX	−0.22	0.55	0.64	0.18	0.06	0.18	17
WQB1	−0.94	1.78	−0.48	−0.57	0.32	0.09	18
WH1	0.54	−1.17	2.17	0.30	−0.65	0.08	19
WH7	−0.12	0.58	−0.25	0.30	−0.94	0.06	20
QH7	−0.40	0.51	0.52	−0.42	0.72	0.06	21
WQHE	−0.82	1.59	−1.70	−0.49	0.32	−0.05	22
QH9	1.13	−2.01	−1.66	2.83	−0.39	−0.08	23
WQH27	−0.52	−0.61	−2.39	0.13	1.88	−0.49	24
QH6	−1.16	−0.25	−1.03	0.51	−0.15	−0.60	25
WQB2	−0.80	−0.59	−1.36	−1.94	0.85	−0.79	26
QH4	−2.05	−0.44	−0.90	1.07	0.80	−0.88	27
WH4	1.42	−4.26	−0.73	−0.45	−0.96	−0.94	28
WQHC	−2.03	−1.52	0.66	−0.17	0.88	−1.15	29
QH8	−1.79	−3.61	0.28	−0.82	0.73	−1.82	30
WQHA	−2.55	−2.93	−0.04	−1.73	−1.77	−2.24	31
WQHQ	−3.17	−2.75	0.02	−1.24	−0.29	−2.26	32
WQC3	−7.59	0.30	0.22	1.31	−0.43	−2.79	33

**Table 7:** Name and number of tested *Xanthoceras sorbifolium* cultivars.

Number	Cultivars Name	Cultivars Code	Germplasm Type	Number	Cultivars Name	Cultivars Code	Germplasm Type	Number	Cultivars Name	Cultivars Code	Germplasm Type
1	Wo qibai No. 1	WQB1	Asexual line	12	Wo qihong No. S	WQHS	Asexual line	24	Qi hong No. 9	QH9	Asexual line
2	Wo qibai No. 2	WQB2	Asexual line	13	Wo qihong No. X	WQHX	Asexual line	25	Qi hong No. 10	QH10	Asexual line
3	Wo qibai No. 3	WQB3	Asexual line	14	Wo qihong No. 27	WQH27	Asexual line	26	Wo hong No. 1	WH1	Asexual line
4	Wo qibai No. 4	WQB4	Asexual line	15	Qi hong No. 1	QH1	Asexual line	27	Wo hong No. 3	WH3	Asexual line
5	Wo qichong No. 3	WQC3	Asexual line	16	Qi hong No. 2	QH2	Asexual line	23	Wo hong No. 4	WH4	Asexual line
6	Wo qihong No. A	WQHA	Asexual line	17	Qi hong No. 3	QH3	Asexual line	28	Wo hong No. 5	WH5	Asexual line
7	Wo qihong No. C	WQHC	Asexual line	18	Qi hong No. 4	QH4	Asexual line	29	Wo hong No. 6	WH6	Asexual line
8	Wo qihong No. D	WQHD	Asexual line	19	Qi hong No. 5	QH5	Asexual line	30	Wo hong No. 7	WH7	Asexual line
9	Wo qihong No. E	WQHE	Asexual line	20	Qi hong No. 6	QH6	Asexual line	31	Wo hong No. 8	WH8	Asexual line
10	Wo qihong No. P	WQHP	Asexual line	21	Qi hong No. 7	QH7	Asexual line	32	Wo hong No. 9	WH9	Asexual line
11	Wo qihong No. Q	WQHQ	Asexual line	22	Qi hong No. 8	QH8	Asexual line	33	Wo hong No. 10	WH10	Asexual line

**Table 8:** Description and grouping of qualitative characters of ornamental *Xanthoceras sorbifolium* cultivars.

Grading	The Color of One-Year Branchlets	Presence of Hair on One-Year-Old Branchlets	Petal Type	Leaf Shape	Curl Degree of Leaflets	The Color of the Lower Part of the Petals at S1	The Color of the Upper Part of the Petals at S3	The Color of the Upper Part of the Petals at S1	The Color of the Lower Part of the Petals at S3	The Color of the Lower Part of the Petals at S2	The Color of the Upper Part of the Petals at S
Grade 1	With green and purplish red	Were hairless	Single petals	Lanceolate	Not curly	Yellow group	White group	White group	Orange group	Yellow group	White group
Grade 1	Purplish red	Were hairy	Double petals	Ovate-lanceolate	Micro curl	Yellow-green group	Red group	Green-yellow group	Orange-red group	Yellow-orange group	Yellow group
Grade 1				Near ovate	Mainly curly	Green-yellow group	Red-violet group	Yellow-green group	Red group	Orange group	Yellow-orange group
Grade 1								Yellow group	Red-violet group	Orange-red group	Orange group
Grade 1										Red group	Red group
Grade 1											Violet group

## Data Availability

All data generated or analyzed during this study are included in this published article.
